# Novel Binding Mode of a Potent and Selective Tankyrase Inhibitor

**DOI:** 10.1371/journal.pone.0033740

**Published:** 2012-03-16

**Authors:** Hakan Gunaydin, Yan Gu, Xin Huang

**Affiliations:** Department of Molecular Structure, Amgen, Cambridge, Massachusetts, United States of America; Medical School of Hannover, United States of America

## Abstract

Tankyrases (TNKS1 and TNKS2) are key regulators of cellular processes such as telomere pathway and Wnt signaling. IWRs (inhibitors of Wnt response) have recently been identified as potent and selective inhibitors of tankyrases. However, it is not clear how these IWRs interact with tankyrases. Here we report the crystal structure of the catalytic domain of human TNKS1 in complex with IWR2, which reveals a novel binding site for tankyrase inhibitors. The TNKS1/IWR2 complex provides a molecular basis for their strong and specific interactions and suggests clues for further development of tankyrase inhibitors.

## Introduction

**Figure 1:**
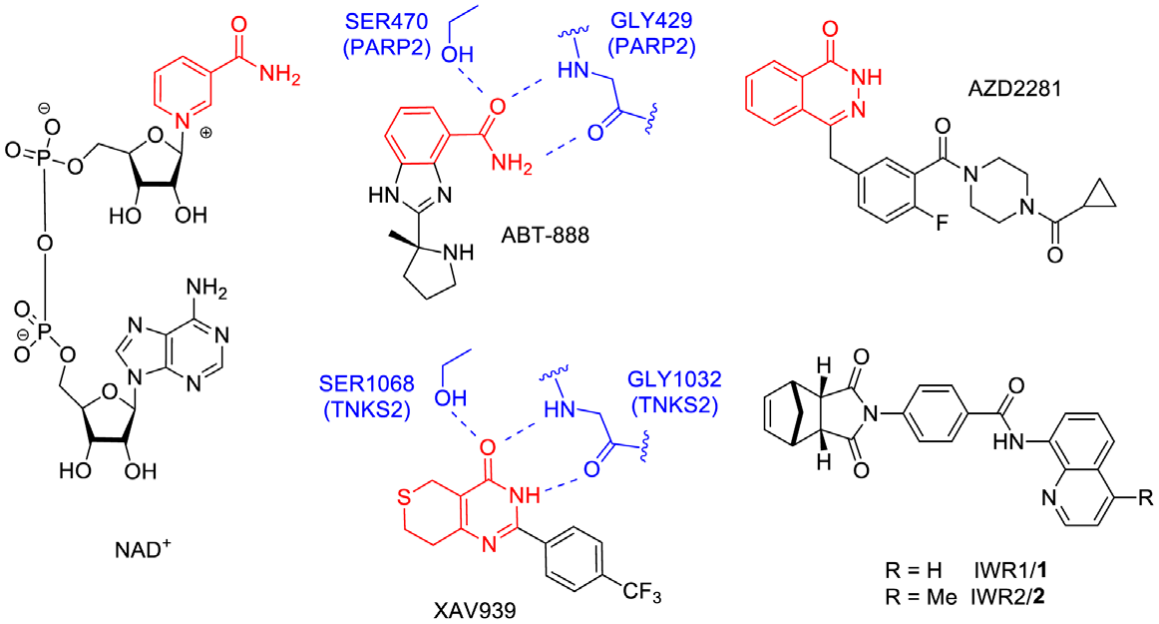
Chemical structures of NAD+, ABT-888, AZD2281, XAV939, IWR1, and IWR2 and the binding modes of ABT-888 and XAV939 to PARP2 and TNKS2. The nicotinamide in NAD+ and the nicotinamide-mimic moieties in ABT-888, AZD2281, and XAV939 are highlighted in red. ABT-888 and XAV939 bind to conserved serine and glycine residues of PARP2 and TNKS2 through three hydrogen bonds. These serine and glycine residues as well as the hydrogen bonds are highlighted in blue.

The two highly homologous human tankyrase isoforms, TNKS1 and TNKS2, are members of the poly ADP-ribose polymerase (PARP) family of 17 proteins that share a catalytic PARP domain [1]. These PARP proteins cleave NAD+ (Figure 1) into ADP-ribose and nicotinamide and transfer the ADP-ribose units onto their substrates, resulting in a post-translational modification referred to as PARsylation. Cellular functions of many PARP proteins remain unknown.

PARP1 and PARP2, the two best characterized family members, are key players in homologous recombination DNA damage response and have been pursued as cancer targets for over a decade [2]. A few PARP1/2 inhibitors such as (R)-2-(2-methylpyrrolidin-2-yl)-1H-benzo[d]imidazole-4-carboxamide (ABT-888) and 4-(3-(4-(cyclopropanecarbonyl)piperazine-1-carbonyl)-4-fluorobenzyl)phthalazin-1(2H)-one (AZD2281) (Figure 1) have shown promising results in clinical trials [3]. They contain functional groups that resemble nicotinamide. Structural studies of PARP inhibitor complexes reveal that these compounds are anchored in the nicotinamide pocket in a very similar manner [4]. Using ABT-888 as a representative example, the nicotinamide oxygen forms hydrogen bonds with both the side chain hydroxyl of Ser470 and the hydrogen NH of Gly429 in PARP2, while one of the hydrogens on the primary amide forms a hydrogen bond with the main chain oxygen of Gly429 in PARP2. In addition, the imidazole of ABT-888 stacks with the side chain of Tyr472 of PARP2.

Recently, tankyrases have gained increased attention as potential drug targets. They were first discovered as factors that regulate telomere homeostasis by modifying the negative regulator of telomere length, TRF1 [5]. Tankyrases also mark axin, the concentration-limiting component of the β-catenin destruction complex, for degradation, and tankyrase inhibition antagonizes the Wnt signal transduction pathway by stabilizing axin and promoting β-catenin degradation [6]. Therefore, inhibition of tankyrase activity appears to be a promising strategy for multiple therapies in the treatment of cancer. So far, two different classes of potent and selective small molecule tankyrase inhibitors, 4-((3aR,4S,7R,7aS)-1,3-dioxo-3a,4,7,7a-tetrahydro-1H-4,7-methanoisoindol-2(3H)-yl)-N-(quinolin-8-yl)benzamide (IWR1) and 2-(4-(trifluoromethyl)phenyl)-7,8-dihydro-3H-thiopyrano[4,3-d]pyrimidin-4(5H)-one (XAV939), have been identified [6], [7]. IWR1 (**1**) inhibits TNKS1 and TNKS2 with IC_50_ of 131 nM and 56 nM, respectively, but does not inhibit PARP1 or PARP2 up to a concentration of 18.75 µM [6]. XAV939 was originally developed as a PARP1/2 inhibitor, albeit a weak one with IC_50_ of 2.2 µM and 0.11 µM for PARP1 and PARP2, respectively, and it was recently reported to be a more potent inhibitor of TNKS1 and TNKS2 with IC_50_ of 11 nM and 4 nM, respectively [6]. As expected, XAV939 binds to the nicotinamide pocket of TNKS2 through interactions similar to those observed in other PARP inhibitor complexes (Figure 1) [8], maintaining the three aforementioned, conserved hydrogen bonds with a serine hydroxyl, as well as the oxygen and NH from a glycine main chain. In this TNKS2 structure, XAV939 cyclic amide behaves as an isosteres for ABT-888's primary amide. There is also a stacking interaction between the pyrimidinone of XAV939 and the Tyr1071 side chain of TNKS2. IWR compounds, however, do not share these features for anchoring in the nicotinamide pocket (Figure 1). It is not clear how these IWR compounds bind to tankyrases and thus the structure-activity relationship for these compounds has been difficult to interpret [9].

Herein, we report a high-resolution crystal structure of the Human TNKS1 catalytic domain in complex with IWR2 (**2**) (PDB code: 4DVI) and describe the structural basis for its potency and selectivity over PARP1 and PARP2. Our structure reveals a novel binding mode for a tankyrase inhibitor and provides a clear explanation for the reported structure-activity relationship of the IWRs, and important clues for the further optimization of these compounds.

## Results and Discussion

**Figure 2:**
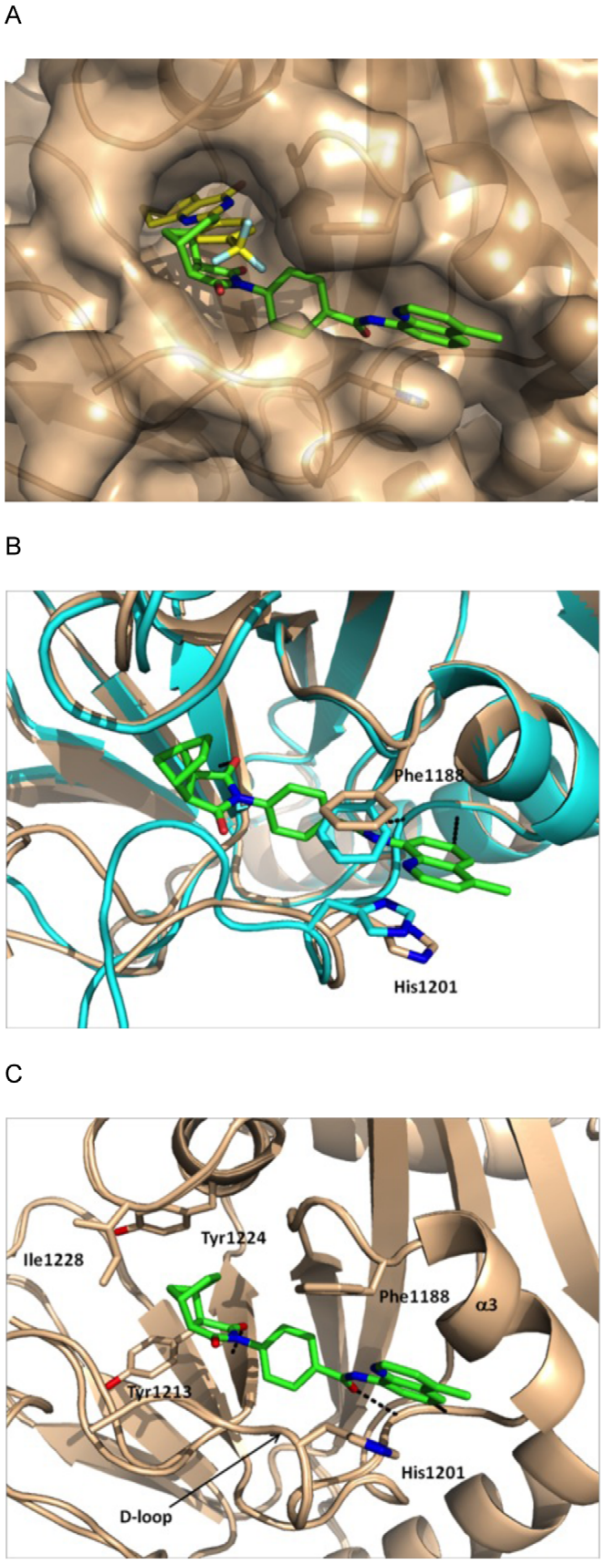
Crystal Structure of the TNKS1/IWR2 complex. (A) Surface representation of TNKS1 (colored in wheat) with IWR2 (colored in green) bound. XAV939 (colored in yellow) from the crystal structure of TNKS2/XAV939 is superimposed to illustrate that IWR2 binds to a different pocket other than the nicotinamide pocket. (B) Superposition of crystal structures of TNKS1/IWR2 (colored in wheat and green) and apo TNKS1 (colored in cyan), with residues Phe1188 and His1201 in sticks, to illustrate the opening of the induced pocket in TNKS1 upon IWR2 binding. IWR2 binds to TNKS1 through three highlighted hydrogen bonds. (C) The induced pocket, showing the hydrogen bond and hydrophobic interactions between IWR2 and TNKS1 residues, colored as in (A).

The crystals of the TNKS1/**2** complex diffracted to 1.9 Å with synchrotron radiation. There are two crystallographically independent TNKS1/**2** complexes in the crystal structure, highly similar to each other (with a backbone rmsd of 0.6 Å). The TNKS1/**2** complex structure reveals that **2** does not bind to the nicotinamide pocket but instead occupies a different pocket (Figure 2A), which is not present in either apo or XAV939 bound tankyrase structures (Figure 2B) [8], [10]. It only becomes available upon the binding of **2** and we thus refer to it as the induced pocket. This induced pocket is created by the movement of Phe1188 of the α3 helix and the D-loop, part of which is disordered in the present crystal structure, away from one another. The binding of **2** to the induced pocket of TNKS1 suggests that IWR compounds are likely non-competitive inhibitors of tankyrases.

In the crystal structure, **2** adopts a conformation in which the central phenyl is almost perpendicular to the norbornyl group and rotated by about 60° away from the plane of the amide group (Figure 2C). There are three hydrogen bonds between **2** and TNKS1. One of the two carbonyl oxygens of the pyrrolidine dione group is hydrogen bonded to the main chain NH of Tyr1213 and the carbonyl oxygen of the amide group is hydrogen bonded to the main chain NH of Asp1198. The CH at the 6-position of the quinoline is also involved in a CH^…^O = C hydrogen bonding interaction with the main chain carbonyl oxygen of Gly1196. Moreover, the quinoline group in **2** engages in hydrophobic interaction with the side chain of Phe1188 and stacking interaction with the side chain of His1201 of the D-loop. The quinoline group is co-planar to the amide group as a result of the intra-molecular hydrogen bond between the quinoline nitrogen and the amide NH.

**Figure 3:**
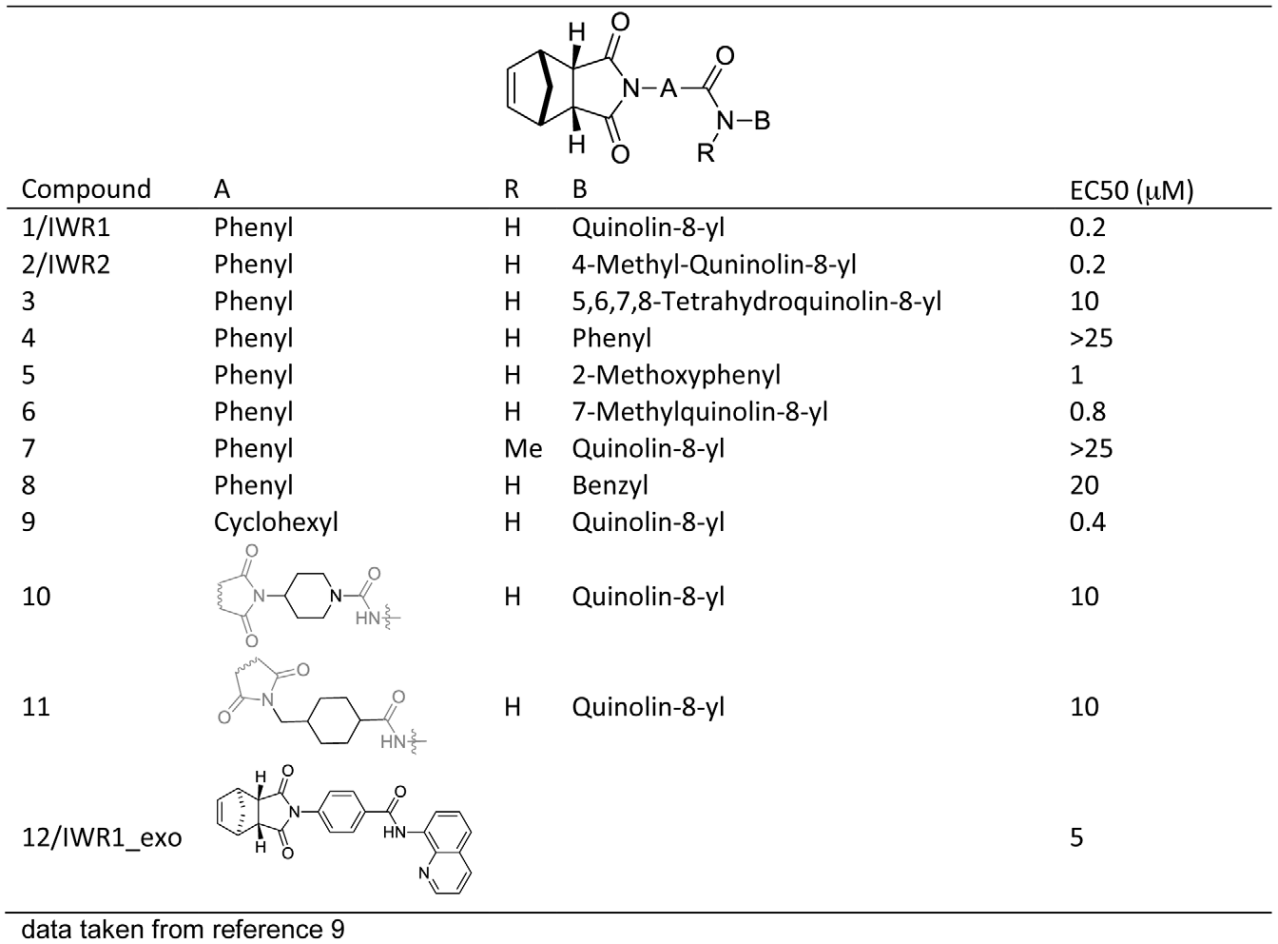
Structure activity relationship of IWR compounds.

Structure-activity relationship studies carried out previously with some of the analogs of **2** in a cellular luciferase-based reporter assay can now be interpreted with the hydrogen bonding and hydrophobic interactions identified from the TNKS1/**2** crystal structure (Figure 3) [9]. The methyl group at the 4 position of the quinoline moiety of **2** does not enhance interaction with TNKS1 and it is not surprising that the reported inhibitory activity of the des-methyl compound, **1**, is similar to that observed for **2**. However, replacement of the quinoline group in **1** with 5,6,7,8 tetrahydroquinoline group (**3**) dramatically changes the conformation and severely diminishes the activity and highlights the importance of the above mentioned CH^…^O = C interaction and the stacking interaction with His1201. Deletion of the pyridine moiety from the quinoline ring (**4**) also leads to loss of the stacking interaction with His1201 and abolishes activity. A methoxy group, on the other hand, is known to engage in or enhance the stacking interaction with aromatic groups [11], thus the addition of 2-methoxy substituent to **4** restores most of the activity (**5**). Quantum mechanical calculations (Figure S1) indicate that introduction of a methyl group to the 7 position of the quinoline does not distort the co-planar conformation of the amide quinoline critical for stacking against the His1201 side chain as much as the methylation of the amide group. Consistent with this analysis, the methylated quinoline analog (**6**) is only 4 fold less potent than **1** while the N-methylated amide analog (**7**) does not have any measurable activity up to a concentration of 25 µM. Similarly, the benzyl amide analog (**8**) needs to adopt a strained conformation in order to engage in a face-to-face stacking interaction with His1201 (Figure S1) and has, as a result, diminished activity. According to quantum mechanical calculations, the saturation of the central phenyl group in **1** does not alter the conformational preferences significantly (Figure S1) and is likely to maintain the important hydrogen bonding and stacking interactions between **1** and TNKS1. There is only a slight loss in activity for the cyclohexyl analog **9**. However, replacement of the central phenyl with a piperidine group would make it energically much less favorable to adopt the conformation observed in the crystal structure (Figure S1). Consistent with our analysis, **10** is 25 fold less active than **9**. In addition, the extension of the middle cyclohexyl group in **9** with an extra methylene atom (**11**) is likely to disrupt the hydrogen bonding interactions and results in significant loss of inhibitory activity. Interestingly, the exo enantiomer of **1** (**12**) is 25 fold less active than the endo enantiomer even though the structural difference between the two enantiomers is very subtle: the spatial swapping of the ethylene moiety with the methylene bridge head converts the endo enantiomer to exo enantiomer. This suggests that the partially positive hydrogen atoms of the ethylene group may not be as well tolerated as the bridgehead methylene group in the pocket created by Tyr1213, Tyr1224, and Ile1228 of TNKS1.

**Figure 4:**
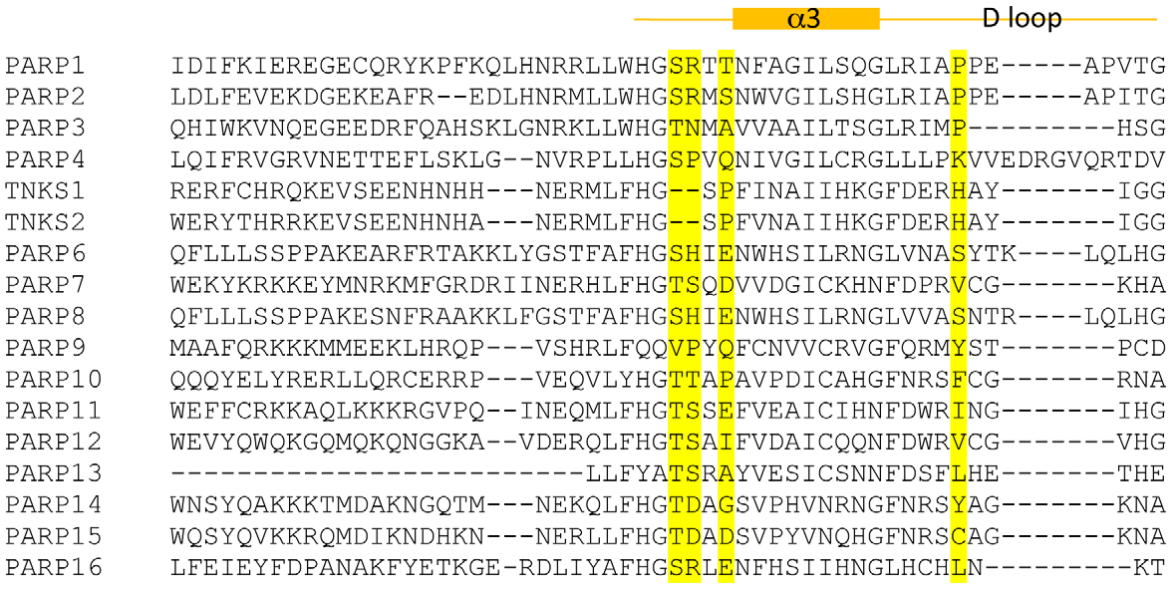
Structure based sequence alignment of TNKS1, TNKS2, and other PARP family members. Key residues Pro1187 (following deletion of two amino acids) and His1201 of the induced pocket in TNKS1 are highlighted, together with their equivalent residues in other PARP proteins, to illustrate the poor conservation of these amino acids.

Inhibitors that bind to the induced pocket of tankyrases possess advantages in terms of chemical space and selectivity. Since the nicotinamide pocket has been well explored for designing PARP inhibitors, it may be challenging to come up with new chemotypes that bind to the nicotinamide pocket for the inhibition of tankyrases. IWRs represent a new class of tankyrase inhibitors that bind to the previously unknown induced pocket and it is likely that other chemotypes may also bind to this induced pocket that maintain the key binding interactions observed for **2**. Residues composing the nicotinamide pocket are highly conserved among all PARP family members, presenting a major challenge for the development of specific tankyrase inhibitors. The regulatory helical domain of PARP1, PARP2, PARP3, and PARP4 immediately N-terminal to the catalytic domain could be used to obtain some selectivity over these PARP proteins as in the case with XAV939 which sterically clashes with the N-terminal helical domain of PARP1, PARP2, PARP3, and PARP4 [8]. This N-terminal helical domain, however, is not conserved in other PARP proteins, making it very difficult to achieve broader selectivity among the PARP family for tankyrase inhibitors. Residues forming the induced pocket of tankyrases, on the other hand, are much less conserved among other PARP family members (Figure 4). For example, the critical His1201 from the D-loop of TNKS1 (His1048 in TNKS2) is not conserved in other PARP proteins; the α3 helix N-terminal to the D-loop is slightly shorter in tankyrases due to the insertion of a proline (Pro1187) and deletion of two amino acids, resulting in a narrower induced pocket. Therefore, one is likely to achieve broader selectivity over PARP family members with compounds that bind to the induced pocket. For example, the selectivity of XAV939 for TNKS1 over PARP2 is only 10 fold whereas the selectivity of **2** is greater than 143 fold [6].

The TNKS1/**2** complex structure and molecular modeling analysis suggest a number of distinct routes to further optimize tankyrase inhibitors that bind to the induced pocket. Preliminary metabolic stability studies indicated enzymatic cleavage of the amide bond to be the primary clearance mechanism for IWRs [9]. It is clear from our crystal structure that the amide quinoline of **2** can be replaced by other more stable moieties that maintain the same hydrogen bonding and stacking interactions. Modifications of the central phenyl group may also generate compounds with more favorable binding geometries. Quantum mechanical calculations suggest that the ∼60° dihedral between the phenyl and amide observed in the crystal structure of **2** results in an intrinsic reduction of potency by approximately 25-fold (∼2 kcal/mol). The pyrrolidine dione group also does not appear optimal for tankyrase binding. One of the two carbonyl oxygens is not involved in hydrogen bonding or any other interaction with the protein and thus could be replaced. In addition, it is also conceivable that the norbornyl group does not interact optimally with the Tyr1213, Tyr1224, and Ile1228 of TNKS1. Furthermore, since the induced pocket is adjacent to the nicotinamide pocket which is unoccupied and unhindered, it may be possible to extend the induced pocket binding tankyrase inhibitors such as **2** into the nicotinamide pocket to gain additional interactions, resulting in even greater potency while maintaining good selectivity due to the specificity of the induced pocket.

IWR compounds may have activity for proteins other than PARP family members; thus, minimizing potential side effects from the off-target interactions is essential for further development of tankyrase inhibitors derived from IWRs. Future studies such as chemical proteomics screens need to be carried out to identify potential unintended targets of these inhibitors.

We note that induced pockets have been observed for other enzymes such as protein kinases. An allosteric binding pocket was reported for a diaryl urea class of highly potent and selective inhibitors against human p38 MAP kinase and the formation of this pocket requires a large conformation change [12]. Improving interactions in this allosteric pocket and establishing additional interactions in the adjacent ATP pocket enhanced the affinity of the inhibitors by 12,000 fold [12]. Imatinib, developed to treat chronic myelogenous leukemia (CML) and gastrointestinal stromal tumor (GIST), binds to similar sites in the human Abl and Kit kinases and shows excellent efficacy and specificity for Abl and Kit [13], [14]. Interestingly, imatinib was found to inhibit strongly (IC_50_ = 80 nM) a non-kinase target, the oxidoreductase NQO2, from a screen carried out to identify off-target proteins [15]. Vemurafenib, developed for the treatment of metastatic melanoma caused by the BRAFV600E mutation, also binds to an induced pocket created by an outward shift of the αC helix [16].

In summary, the present structure reveals a novel binding mode for tankyrase inhibitors and, in conjunction with molecular modeling analysis, provides insights into the molecular basis for the key interactions between IWRs and tankyrases. In addition, it explains the structure activity relationship of the IWRs and will be important for further optimization of tankyrase inhibitors.

## Materials and Methods

Human TNKS1 (1104–1314) with a C-terminal His_6_ tag was cloned into the PET28a vector and expressed in *E. Coli* Rosetta (DE3). The culture was grown in TB media at 37°C until OD_600_ reached ∼2. The culture was then cooled to 18°C and induced by addition of 0.5 mM IPTG. Expression was allowed to continue overnight and cells were harvested by centrifugation. The resulting cell pellet was resuspended in lysis buffer (25 mM Hepes, 300 mM NaCl, 2.5 mM BME, pH 8.0) supplemented with 0.8% Protease Inhibitor Cocktail (Sigma). The cells were lysed by Microfluidizer (Microfluidics) and cell debris was removed by centrifugation (19000× g, 90 min, 4°C). The supernatant was incubated with Talon Metal Affinity resin (Clontech) overnight at 4°C before loaded onto a column. The Co++ Talon resin was washed with a lysis buffer containing 5 mM Imidazole. TNKS1His_6_ was then eluted with a lysis buffer containing 60 mM Imidazole. The TNKS1His_6_ protein was further purified in gel filtration buffer (25 mM Hepes, 200 mM NaCl, 14 mM BME, 5% glycerol, pH 8.0) by size exclusion chromatography using Superdex 200 (GE Healthcare).

The TNKS1/IWR2 complex was obtained by incubating TNKS1His_6_ at 10 mg/ml with IWR2 (commercially available from AKos) in 2-fold molar excess for 30 minutes at 4°C. Crystals of TNKS1/IWR2 were obtained at 4°C in hanging drops by mixing 0.5 µL of TNKS1/IWR2 complex with 0.5 µL of well solution containing 100 mM MES pH 6.0, 0.2 M or 0.4 M Di-Ammonium Tartrate, 12.5–25% PEG3350. Plate shaped crystals appeared overnight and grew to maximum size in a few days. These crystals belong to the spacegroup P2_1_2_1_2_1_ with unit cell parameters of a = 41.47, b = 77.94, c = 146.54 Å. Paratone-N mineral oil was used as cryo protectant and diffraction data were collected on beamline 5.0.1 at the Advanced Light Source (ALS), Berkeley, CA and processed with HKL2000. The TNKS1/IWR2 complex structure was solved by molecular replacement with AMoRe using the apo TNKS1 structure (2RF5) as the template. Model building was carried out with QUANTA and refinement was done using CNX. Details on data processing and refinement statistics are given in Table S1.

## Supporting Information

Figure S1 **Quantum mechanical calculations were done at the B3LYP/6-31G(d) level of theory using the software package Gaussian 03.** The dihedral energy scan calculations for the dihedrals highlighted were performed with 10 degree increments for the scanned dihedral and all the other dihedrals, angles and distances were allowed to relax during the calculations. Single point solvation energies were computed for the final geometries at the same level of theory using the CPCM solvation method and the UAKS cavity model. In each case, the rotated dihedral is highlighted.
(DOC)

Table S1 **Data Collection and refinement statistics for TNKS1/IWR2 structure.**
(DOC)
