# Supplementary figures and images for: Novel Binding Mode of a Potent and Selective Tankyrase Inhibitor
Source: PLoS One. 2012 Mar 16;7(3):e33740. doi: 10.1371/journal.pone.0033740 (PMC3306292; doi:10.1371/journal.pone.0033740)

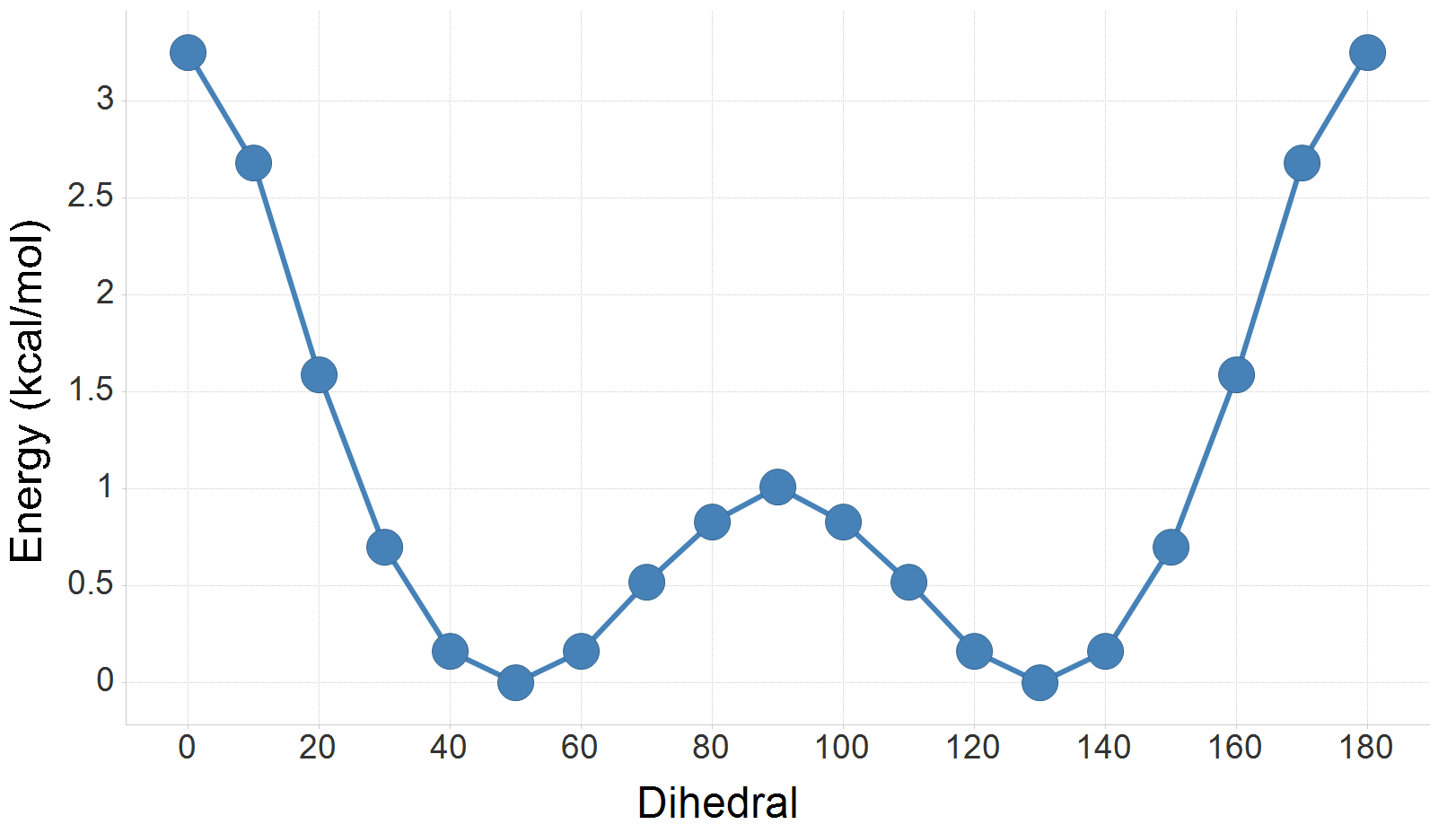


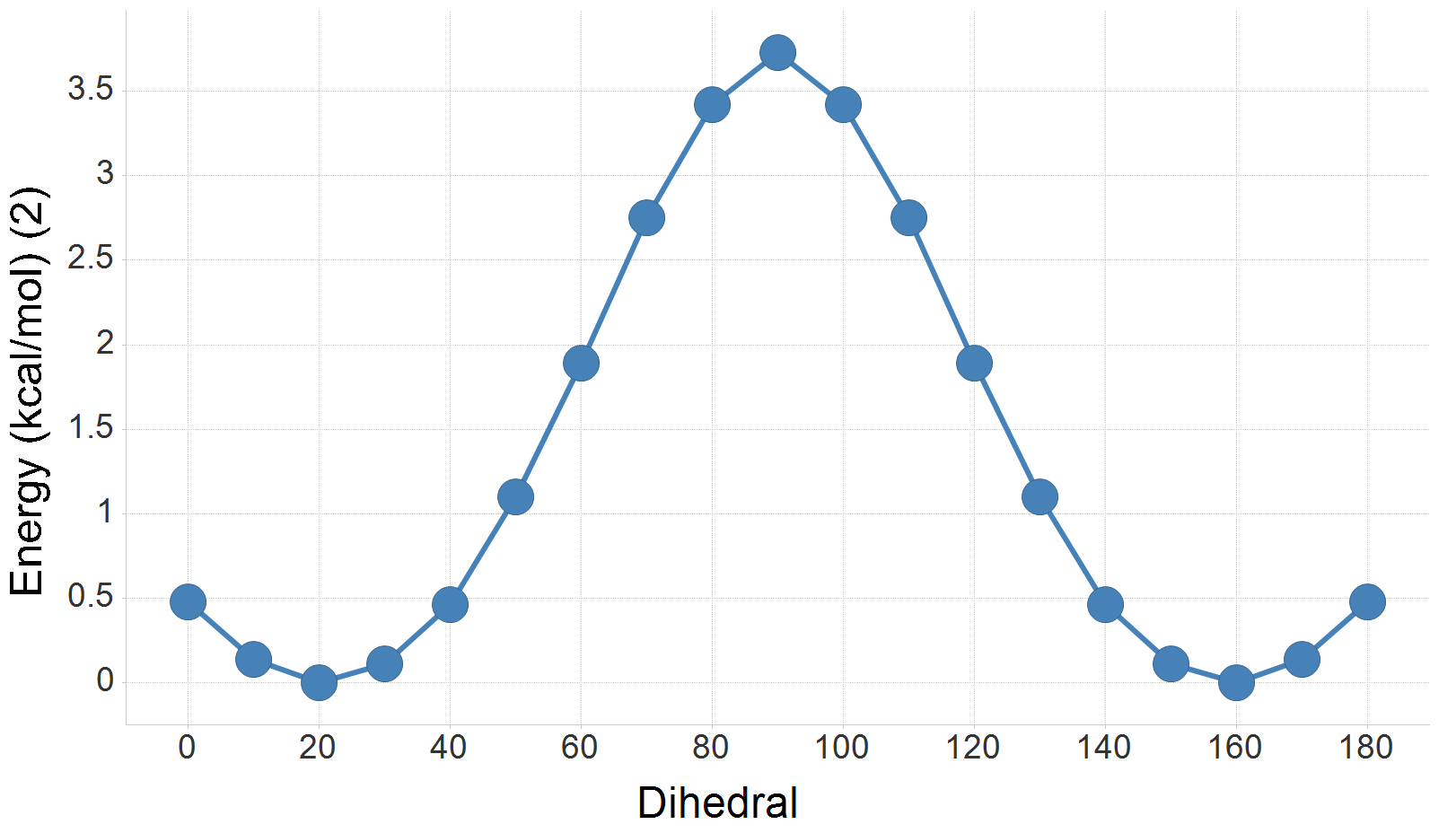


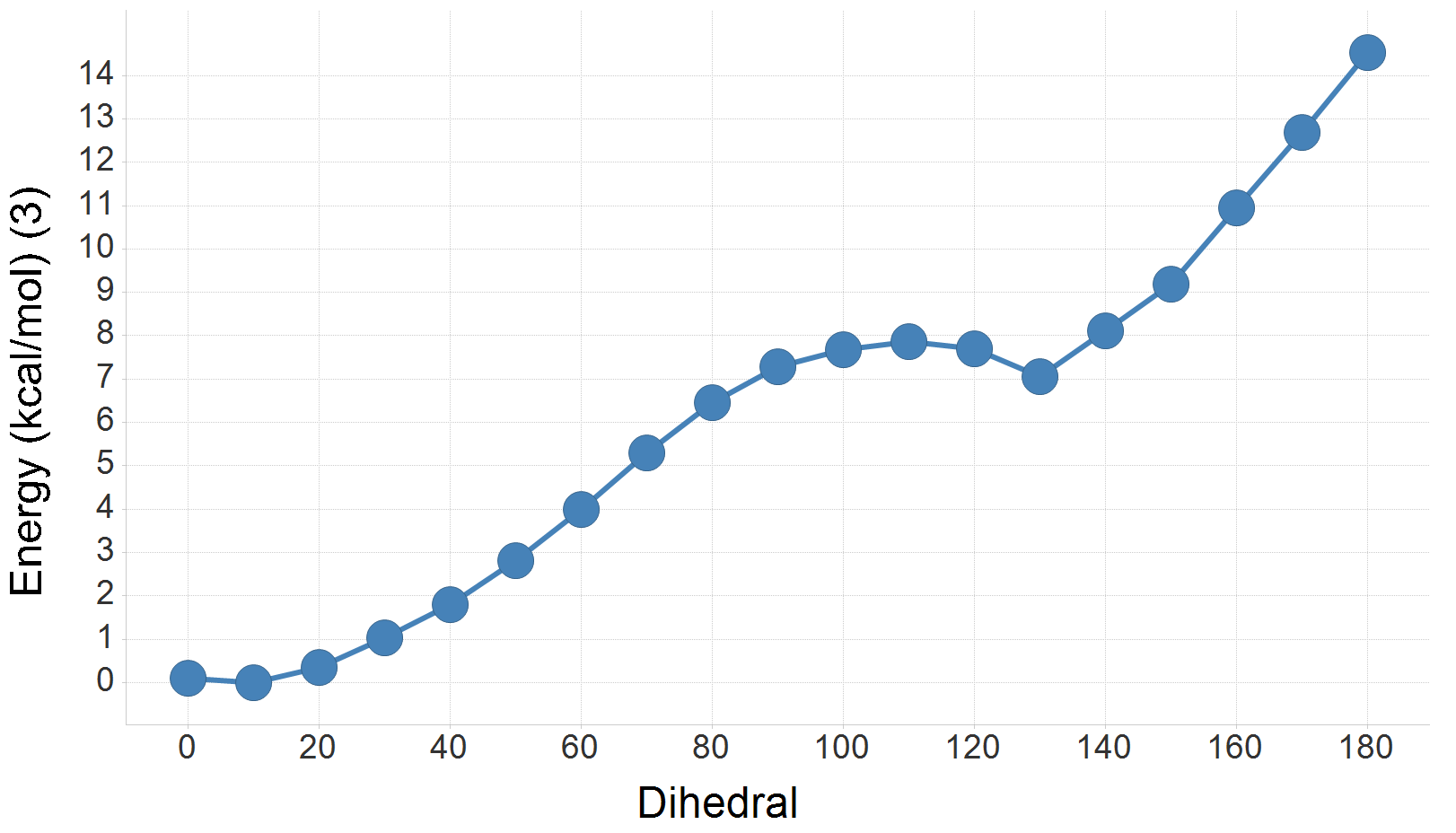


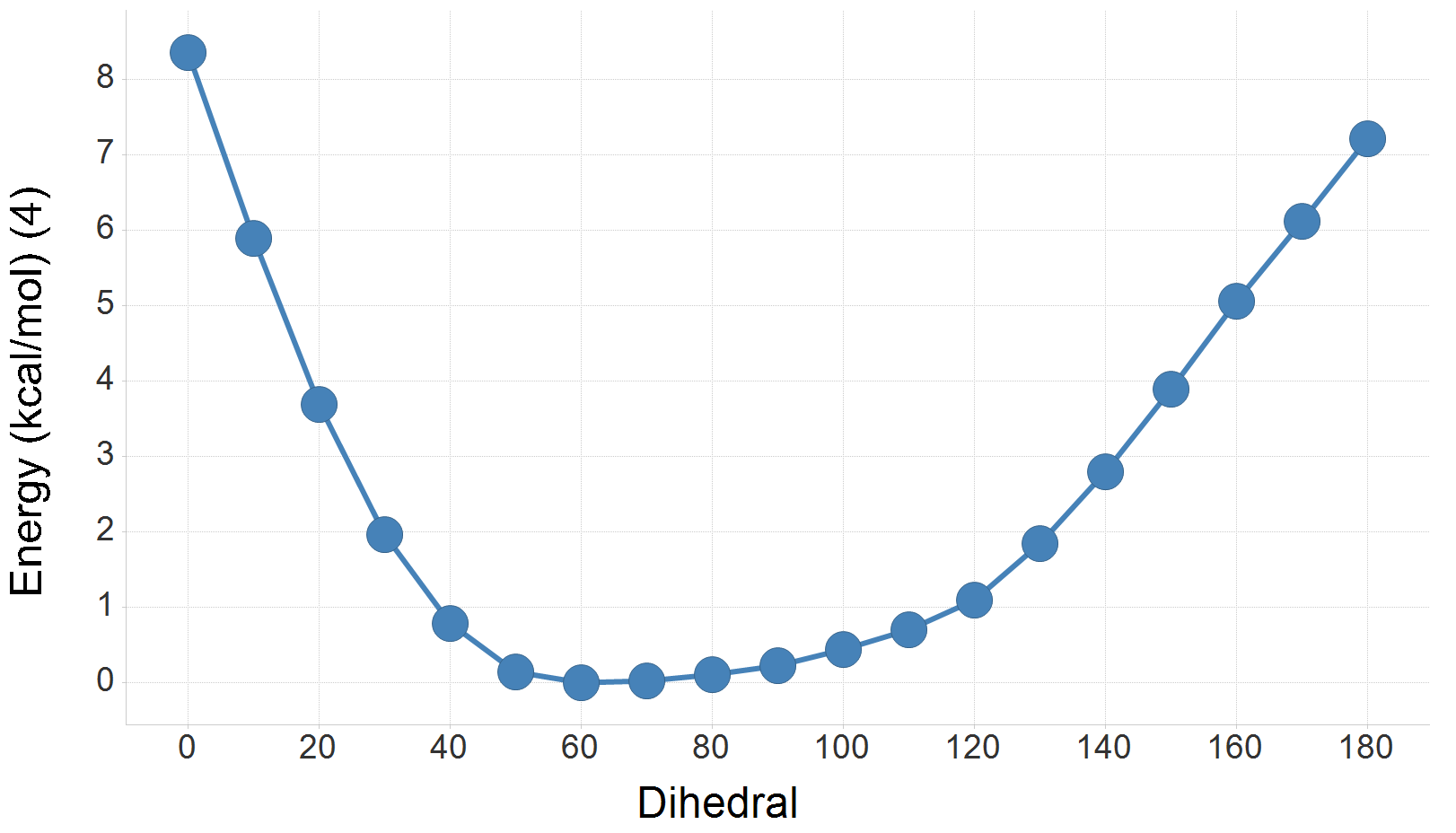


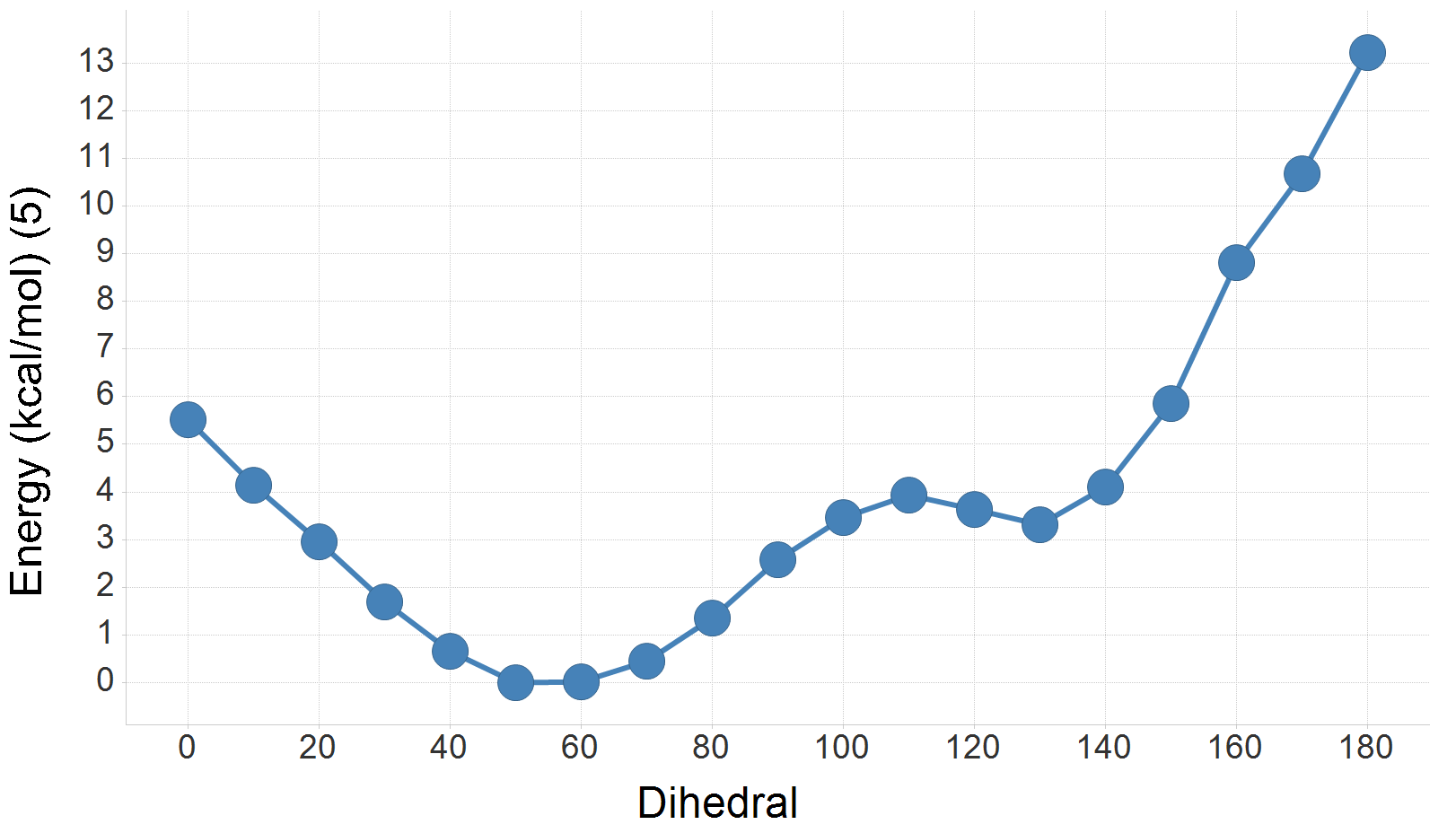


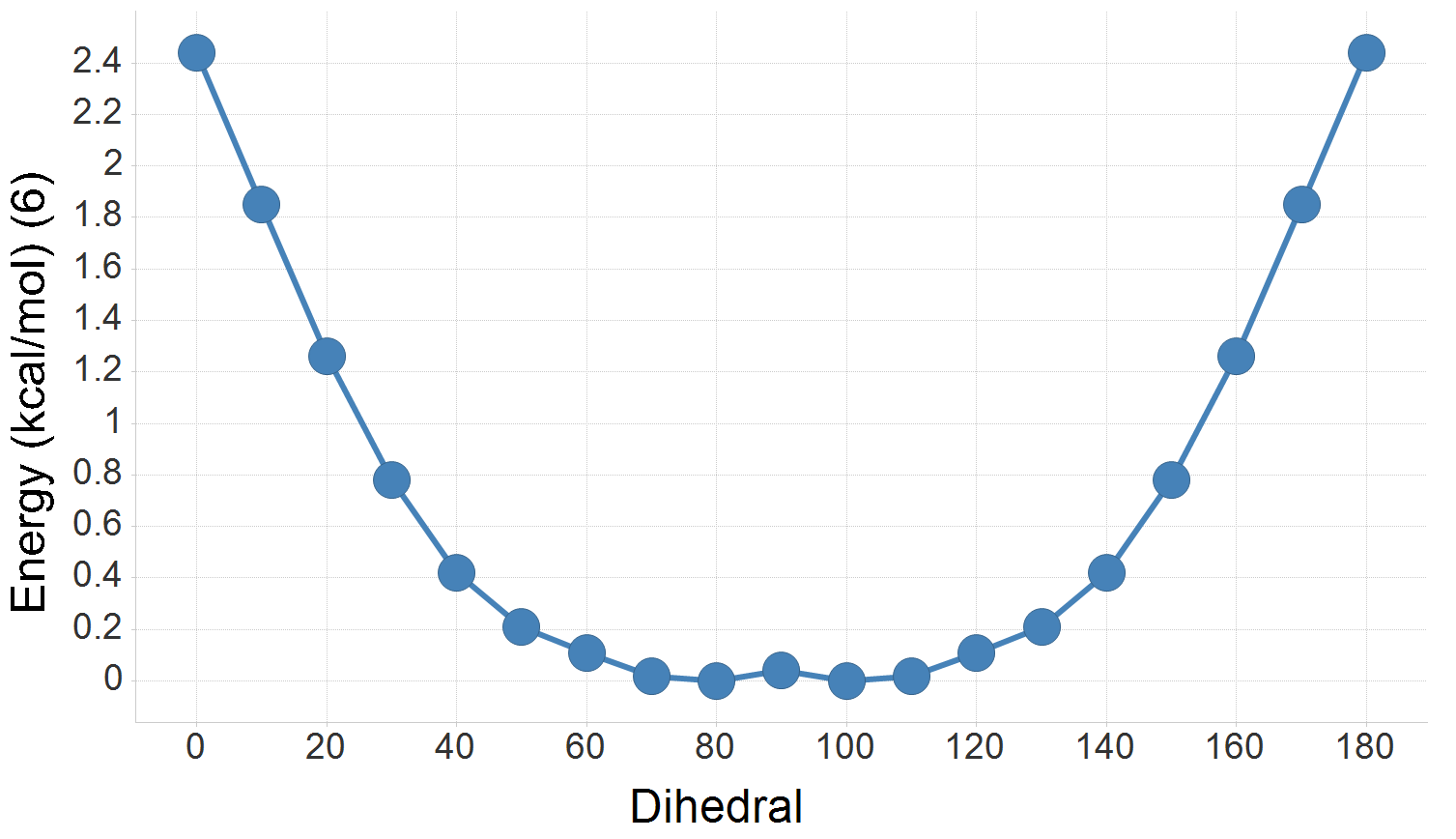


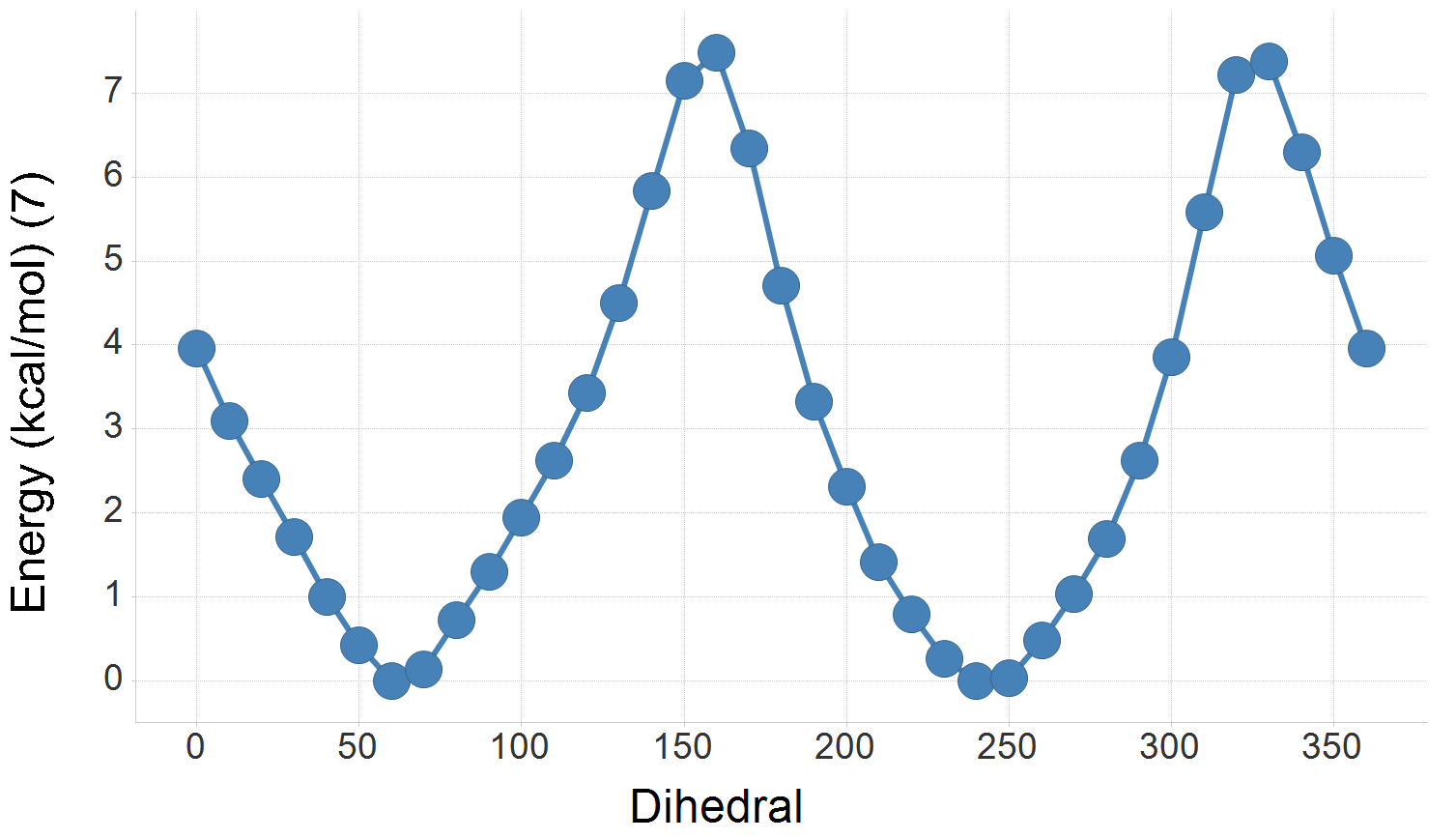


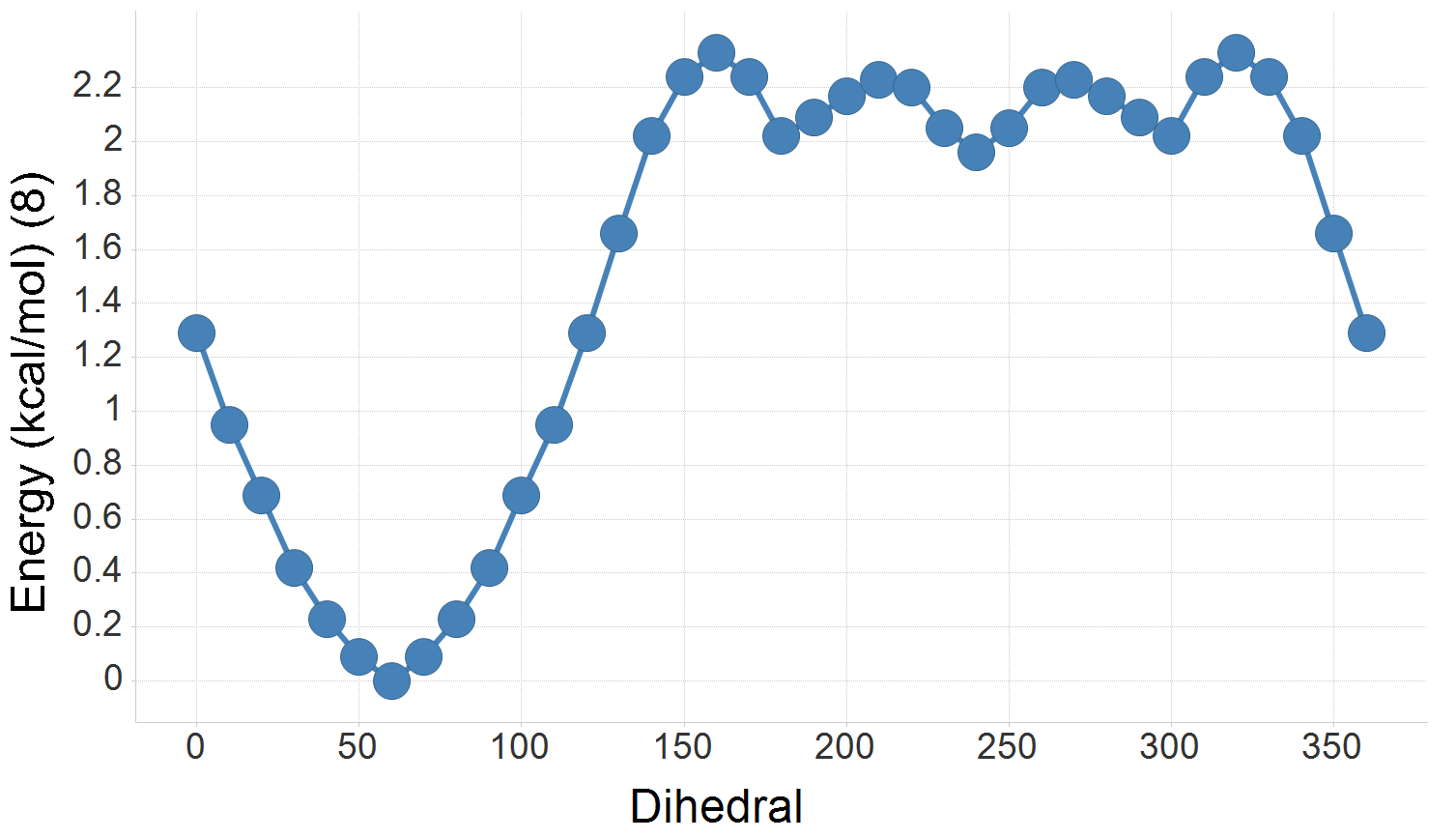


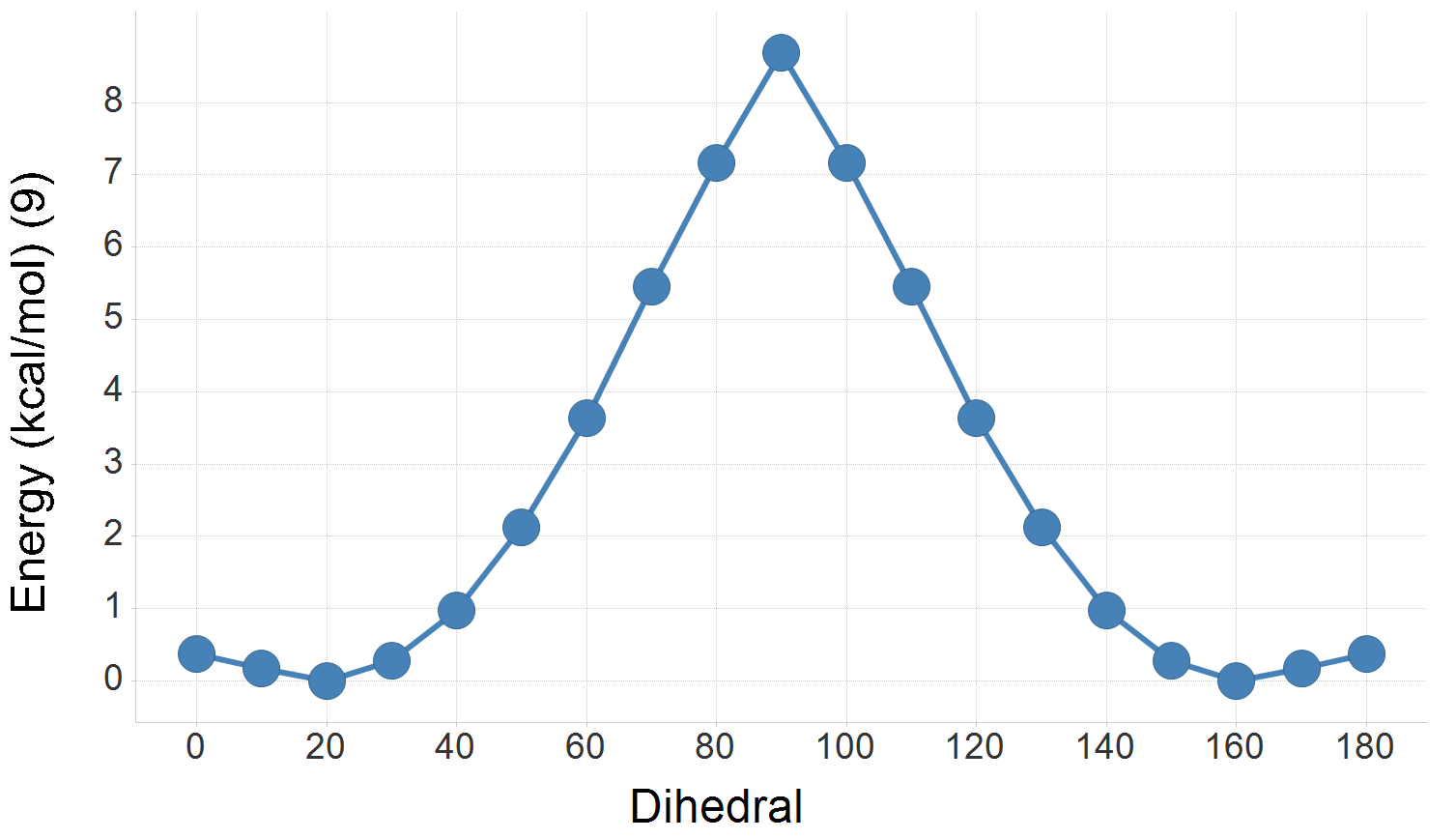

Supplement: Figure S1 — Quantum mechanical calculations were done at the B3LYP/6-31G(d) level of theory using the software package Gaussian 03. The dihedral energy scan calculations for the dihedrals highlighted were performed with 10 degree increments for the scanned dihedral and all the other dihedrals, angles and distances were allowed to relax during the calculations. Single point solvation energies were computed for the final geometries at the same level of theory using the CPCM solvation method and the UAKS cavity model. In each case, the rotated dihedral is highlighted. (DOC) [file pone.0033740.s001.doc]
