# Supplementary material for: Novel Binding Mode of a Potent and Selective Tankyrase Inhibitor
Source: PLoS One. 2012 Mar 16;7(3):e33740. doi: 10.1371/journal.pone.0033740 (PMC3306292; doi:10.1371/journal.pone.0033740)
Supplement: Table S1 — Data Collection and refinement statistics for TNKS1/IWR2 structure. (DOC) [file pone.0033740.s002.doc]

|  | TNKS1/IWR2 | |
| --- | --- | --- |
| Space group | P2_1_2_1_2_1_ | |
| Unit cell a, b, c (Å) | 41.47, 77.94, 146.54 | |
| Mol/ASU | 2 | |
| Wavelength (Å) | 0.97740 | |
| Resolution (Å) | 1.90 | |
| R_merge_ (%) | 10.5 (79.0) | |
| Reflections (total/unique) | | 258747/37889 |
| Completeness (%) | 99.9 (99.4) | |
| Protein atoms | 3268 | |
| Inhibitor atoms | 64 | |
| Water molecules | 312 | |
| R_work_/R_free_ (%) | 24.7/25.8 | |
| Rms deviations |  | |
| Bond length (Å) | 0.016 | |
| Bond Angle (⁰) | 1.85 | |

Values in parentheses are for the highest resolution shell.
